# Identification of Novel Adhesins of *M. tuberculosis* H37Rv Using Integrated Approach of Multiple Computational Algorithms and Experimental Analysis

**DOI:** 10.1371/journal.pone.0069790

**Published:** 2013-07-29

**Authors:** Sanjiv Kumar, Bhanwar Lal Puniya, Shahila Parween, Pradip Nahar, Srinivasan Ramachandran

**Affiliations:** Functional Genomics Unit, Council of Scientific and Industrial Research -Institute of Genomics and Integrative Biology (CSIR-IGIB), New Delhi, India; University of Delhi, India

## Abstract

Pathogenic bacteria interacting with eukaryotic host express adhesins on their surface. These adhesins aid in bacterial attachment to the host cell receptors during colonization. A few adhesins such as Heparin binding hemagglutinin adhesin (HBHA), Apa, Malate Synthase of *M. tuberculosis* have been identified using specific experimental interaction models based on the biological knowledge of the pathogen. In the present work, we carried out computational screening for adhesins of *M. tuberculosis*. We used an integrated computational approach using SPAAN for predicting adhesins, PSORTb, SubLoc and LocTree for extracellular localization, and BLAST for verifying non-similarity to human proteins. These steps are among the first of reverse vaccinology. Multiple claims and attacks from different algorithms were processed through argumentative approach. Additional filtration criteria included selection for proteins with low molecular weights and absence of literature reports. We examined binding potential of the selected proteins using an image based ELISA. The protein Rv2599 (membrane protein) binds to human fibronectin, laminin and collagen. Rv3717 (N-acetylmuramoyl-L-alanine amidase) and Rv0309 (L,D-transpeptidase) bind to fibronectin and laminin. We report Rv2599 (membrane protein), Rv0309 and Rv3717 as novel adhesins of *M. tuberculosis* H37Rv. Our results expand the number of known adhesins of *M. tuberculosis* and suggest their regulated expression in different stages.

## Introduction

Tuberculosis (TB) caused by *M. tuberculosis* (Mtb) continues to be a ravaging disease. In the year 2010, about 8.8 million incidences were reported and 1.1 million people died due to tuberculosis and an additional 0.35 million deaths were due to HIV-associated tuberculosis [1]. However, recent reports display a downward trend in the total number of tuberculosis cases since 2006. This change of pattern is also reflected in the number of deaths due to TB [1]. One of the key features contributing to the success of this pathogen is its unique characteristic lipid rich cell wall [2]. The mycobacterial cell wall envelope is thick, rigid, waxy and consists of inner lipid bilayer plasma membrane. The cell wall is formed by peptidoglycan-arabinogalactan polymers in periplasmic space with outer lipid enriched in mycolic acids covalently linked to the arabinogalactan layer [3]. This elaborate structure renders it nearly impermeable to many conventional antimicrobial drugs thereby limiting the number of effective agents against tuberculosis [4].

Pathogenic bacteria express adhesins, which help in bacterial attachment to the host cell receptors and aid in colonization. Adherence of *M. tuberculosis* to respiratory epithelium induces membrane perturbation and the formation of membrane extensions enable bacterial adherence to respiratory epithelium [5]. Characterization of *M. tuberculosis* attachment to respiratory mucosa led to the identification of surface exposed heparin-binding hemagglutinin adhesin (HBHA), which is required for bacterial attachment in lungs and in extrapulmonary dissemination of the bacteria [6]. A laminin binding protein (LBP) was also identified, which is involved in cytoadherence through recognition of laminin [7].

In a study by Samanich *et al.* (2001) [8] and Singh *et al.* (2005) [9] antibodies against malate synthase were identified in TB patients at different stages of the active disease. They reported that 90% of patients with subclinical tuberculosis had antibodies against malate synthase. Earlier, malate synthase was regularly identified in culture filtrates of mid log phase cultures of *M. tuberculosis* [8]. Recently Kinhikar *et al.* (2006) [10] showed that malate synthase binds to human extracellular matrix proteins laminin and fibronectin and it is anchored on cell wall by an undefined mechanism. Therefore malate synthase is classified as ‘anchorless adhesin’. There are other similar examples of anchorless adhesins reported in the literature such as PavA and Eno in *Streptococcus pneumoniae* [11], FBP54, SEN and SDH in *Streptococcus pyogenes* [11], [12] and Eap in *Staphylococcus aureus* [13]. Another adhesin, a 19 kDa lipoprotein antigen is a major component of the cell wall [14]. This protein was identified while probing for Mtb adhesins with affinity for macrophages. The 19-kDa lipoprotein was found preferentially bound to THP-1 macrophage-like cells and as with other adhesins, this protein was also found to be located on the cell-wall [15].

The cell surface glycoprotein Apa was identified as an adhesin while exploring the possible mechanism of the binding of Mtb to pulmonary surfactant proteins PSP-A [16] and PSP-D [17], the membrane-associated macrophage mannose receptor [18], and dendritic cell-specific intercellular adhesion molecule-3 grabbing non-integrin (DC-SIGN) facilitating their entry. Formerly considered as secreted, Apa has also been shown to be associated with cell wall for long enough to aid attachment to PSP-A [19]. Another protein Cpn60.2 is believed to be involved in bacterial pathogenicity and is essential for cell viability [20]. It appears to be necessary to facilitate efficient bacterial association with macrophages [21].

In each of these cases, adhesins were identified in an interaction model based on the biological knowledge of *M. tuberculosis*. Characterization of these adhesins has illuminated the interaction partners underlying the bacteria-host cell adhesion process. Alternative approaches not based on these models could lead to discovery of other mycobacterial adhesins, which could not only enrich our understanding but also provide us with additional potential vaccine candidates. Such an approach has so far not been applied in *M. tuberculosis*. In recent years the development of reverse vaccinology pioneered by Rappuoli [22] uses computer algorithms to screen for potential vaccine candidates in the initial steps. The algorithm SPAAN [23] is being used for identification of adhesins in the initial stage in many instances [24]–[26]. We used SPAAN for identifying adhesins in *M. tuberculosis*. Further we used an integrative approach by applying additional algorithms, which predict subcellular localization. We report the identification of a cell wall hydrolase adhesin, and two additional adhesins with propensity to bind to extracellular matrix proteins, namely, fibronectin, laminin and collagen. Together these data provide new insights into the system of adhesins of *M. tuberculosis*.

## Materials and Methods

### Selection of novel potential adhesins of *M. tuberculosis*

Protein sequences encoded in the whole genome of *M. tuberculosis* H37Rv were screened for identifying probable adhesin proteins. A selection pipeline was developed for identification of novel potential adhesins through screening of all the protein sequences. The selection criterion included probability of a protein being an adhesin or adhesin-like protein predicted by SPAAN [23] at a threshold of P_ad_>0.65, which is slightly lower than the recommended P_ad_>0.7 because the HBHA adhesin of *M. tuberculosis* had P_ad_ value of 0.6763. In order to reduce the likelihood of acquiring potentially false positives due to lowering of threshold, we analyzed the protein sequences through subcellular location prediction algorithms for obtaining additional claims on their features. Surface localization was predicted using sub-cellular localization prediction algorithms LOCTree [27], PSORTb [28] and SubLoc [29].

**Figure 1:**
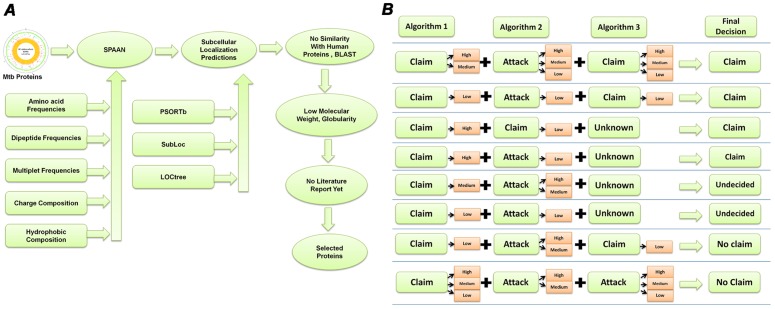
Selection of target proteins. A. The selection pipeline used for investigating computationally predicted adhesins. B. Logic table for combining the results from predictions using multiple algorithms. Predictions from various algorithms were considered for the hypothesis of “adhesin and surface localized” in the forms of Claims (supporting) and Attacks (not-supporting) associated with their confidence of predictions in three categories, High, Medium and Low. Several cases could not be resolved and they remained “undecided” in our protocol.

Because adhesins were our focus, we chose extracellular prediction as ‘claim’ whereas all other predictions corresponded to ‘counter claims’ or ‘attacks’. The logic table for combining the results from individual algorithms is shown in Figure 1B. The cases where a claim wins or loses are displayed. There were a few cases in which the prediction from all three algorithms agreed, but there were several other cases in which the prediction results varied between algorithms. Therefore we used an argumentation based approach for rationalized selection [30]. The confidence levels of predictions in the scale of 10 were classified arbitrarily in three categories 0–3: low, 3–6: medium and >6: high. The ‘claim’ and ‘attacks’ were weighed and the final result was declared as either ‘claim’ or ‘no claim’. In cases of either low confidence prediction by all the algorithms or equally confident claims and attacks, the results were declared as ‘undecided’.

For example, according to the logic table in the cases where a high or medium or low claim from one algorithm is supported by either a high or medium claim by another algorithm, then, even if an ‘attack’ of any confidence occurs by a third algorithm, claim wins. On the other hand if a claim of low confidence by even two algorithms is attacked at high or medium confidence, then the claim looses and the result is declared as ‘no claim’. Also, in cases where a claim of any confidence by an algorithm is attacked by two algorithms of which one of them is an attack of high or medium confidence then the claim looses and the result is declared as ‘no claim’. Other cases presented in the logic table are straight forward. Subsequently, filtration criteria were applied in favor of predicted ‘globular proteins’, low molecular weight (<100 kDa) and absence of any ‘literature reports’. The full list of the results is given in Table S1. BLAST [31] was used for similarity search and identification of orthologues. The TMHMM Server v. 2.0 (Hidden Markov Model for predicting transmembrane helices) [32] was used for predicting the transmembrane topology of proteins. SignalP 3.0 [33] was used for predicting presence of signal peptides. Search using Conserved Domain Database (CDD) [34], Pfam [35] and InterProScan [36] signature were used for predicting conserved domains and classify sequences into protein families.

### Phylogenetic analysis

The protein Rv3717 has N-acetylmuramoyl-L-alanine amidase domain. For phylogenetic analysis, we selected the experimentally characterized N-acetylmuramoyl-L-alanine amidases and those whose crystal structures are available in Protein Data Bank (PDB) database. The protein sequence of Rv3717 and its orthologues in different bacterial species were aligned using MUSCLE [37] with default parameters. The following protein sequences (Uniprot IDs) were selected for phylogenetic analysis: cwhA (P81717) from *Achromobacter lyticus*, pXO2-42 (Q9RMZ0) from *Bacillus anthracis*, cwlL (P36550) from *Bacillus licheniformis*, lytC (Q02114) and cwlC (Q06320) from *Bacillus subtilis*, amiB BH08710 (Q6G3B2) from *Bartonella henselae*, amiB (P57638) from *Buchnera aphidicola*, ampD (P82974) from *Citrobacter freundii*, 38.4 kDa protein (P18020) and cwlD (Q0SQI1) from *Clostridium perfringens*, Endolysin (B6SBV8) from *Clostridium* phage, ampD (P82973) from *Enterobacter cloacae*, NAM-LAA Amidase (P00806) from *Enterobacteria* phage T7, amiA (P36548), amiB (C9QTQ6), amiC (E0IWK5) and amiA (E0J1N3) from *E. coli*, amiB (P44493) from *Haemophilus influenzae*, NAM-LAA Amidase (Q38135) from *Lactococcus* phage r1t, PLY511 (Q38653) from *Listeria* phage A511, NAM-LAA Amidase (P85143) from *Lysobacter* sp., ripA (O53168) and Rv3717 (I6Y4D2) from *M. tuberculosis* H37Rv, amiC (Q9K0V3) and NMB1085 (Q9JZE9) from *Neisseria meningitidis*, cwlB (G0W058), cwlV (Q9LCR3) and cwlU (Q9LCR4) from *Paenibacillus polymyxa*, PAL (O03979) from *Pneumococcus* phage Dp-1, RC0497 (Q92IC3) from *Rickettsia conorii*, amiB (P26366) from *Salmonella typhimurium*, sle1/aaa (P0C1U7) from *Staphylococcus aureus*, atlE (O33635) from *Staphylococcus epidermidis*, sle1 (Q4L3C1) from *Staphylococcus haemolyticus*, sle1 (Q49UX4) from *Staphylococcus saprophyticus*, lytA (P06653) from *Streptococcus pneumonia*.

Phylogenetic analysis was carried out using the PHYLIP 3.69 package [38]. Pair wise sequence distance matrix was computed using Henikoff/Tillier PMB (Probability Matrix from Blocks) matrix [39] in the Protdist program and trees were constructed using Fitch with global rearrangements. Confidence levels for the bifurcating branches were obtained by bootstrap using Seqboot program [38]. Bootstrapping was performed 1000 times. Trees were drawn using Drawtree [38]. The final image was generated using TreeGraph2 [40].

### Protein Expression and purification

All the reagents used were of ExelaR grade. T4 ligase, restriction enzymes were from New England Biolabs (NEB), USA. The vector pET28a was from Novagen, USA. *M. tuberculosis* H37Rv genomic DNA was used as template for PCR amplification of the desired genes. Initially, cloning was done by PCR amplification of the entire open reading frame (ORF). We experienced insurmountable problems in obtaining expression of protein in soluble form for further analysis. Expressed protein was found in inclusion bodies and it was not possible to purify sufficient amount of protein in soluble form. We then attempted purification under denaturing conditions. Purification under denaturing conditions using urea as denaturing agent was accompanied by uncertainty of refolding of the protein back to its native state. Various *E. coli* strains, which could express toxic membrane proteins such as *E. coli* C43 (DE3), C41 (DE3) (Lucigen, USA), Rosetta (Novagen, Germany), BL21 (DE3) pLysS (Invitrogen, USA) were used for expression [41]. Despite repeated attempts we could not arrive at a reproducible method that yielded refolded protein in sufficient amount. Finally, we chose to remove the initial hydrophobic regions in the selected genes, which either encoded for signal peptide or for transmembrane helix and cloned the rest of the ORF and expressed the protein and purified for further analysis. Primers used in the present study are listed in Table 1. Gene specific primers were designed based on the gene sequences from Tuberculist [42]. The part ORF was cloned in pET28a vector at the *BamHI* and *XhoI* sites. The expression was checked in *E. coli* C41 (DE3).

**Table 1:** List of the primers used in the present study.

ORF	Initial amino acids removed	Orientation	Primers	Restriction Enzyme
*Rv0309*	29	Forward Primer	5′ – AGC CTG GCC GGA TCC AAC CCG TGG TTC GCG – 3′	*BamHI*
		Reverse Primer	5′ –CGT CGC ACA GTT CTC GAG CGA GGT CCG GGG – 3′	*XhoI*
*Rv2599*	29	Forward Primer	5′ – ATC ACG CTG GGA TCC AGG GAC GTT GGC TCG – 3′	*BamHI*
		Reverse Primer	5′ –ATA CAT GAC TCT CTC GAG TGC GTC ATC GCC – 3′	*XhoI*
*Rv3717*	24	Forward Primer	5′-ACC CCC GCC GGA TCC GCC GGC ATG GTC GTC-3′	*BamHI*
		Reverse Primer	5′-GCG CGG TGG GGG CTC GAG CTG TGT GCG GGG -3′	*XhoI*

Briefly, recombinant plasmids were prepared in *E. coli* DH5α strain and transformed into *E. coli* C41 (DE3) strain for expression. The transformed cells were grown at 37°C till optical density at 600 nm (OD_600_) reached 0.6 in Terrific Broth (HiMedia, India). The culture was then chilled immediately to 16°C for one hour and then induced with 0.25 mM isopropylthiogalactopyranoside (IPTG) for 12 hours at room temperature (25°C) with constant shaking. Subsequently, the cells were harvested by centrifugation at 6000×g for 10 minutes at 4°C. The pellet was resuspended in sonication buffer (20 mM Tris-HCl (pH 7.9), 500 mM NaCl, 5 mM imidazole, 1 mM phenylmethylsulphonylfluoride (PMSF)). Lysozyme (Hen egg white, Sigma, USA) was added at 1 mg/ml and incubated on ice for one hour. After passing the suspension five times through a 0.8 mm needle syringe the cells were disrupted by sonication (Sonic Vibra-Cell) for 5 minutes with repeated cycles at 40% amplitude of 30 seconds duration followed by 30 seconds rest on ice. The cell lysate was then centrifuged at 29000×g for 30 minutes at 4°C to remove cell debris.

A portion of expressed protein was found soluble by electrophoresis of supernatant and pellet fractions on 12% SDS–PAGE gel [43] and western blot analysis [44] using anti-6×Histidine antibodies conjugated to horseradish peroxidase (HRP) (Qiagen, Germany). Purification of His-tagged protein was carried out by affinity chromatography with Nickel–Nitrilotriacetate (Ni–NTA) resin column (Qiagen, Germany). The soluble fraction was incubated with 2 ml of Ni–NTA resin pre-equilibrated with equilibration buffer (20 mM Tris–HCl, pH 7.9, 500 mM NaCl) overnight at 4°C. The supernatant was loaded onto the column and washed with 10 column volumes of wash buffer-1 (20 mM Tris–HCl, pH 7.9, 500 mM NaCl, 5 mM imidazole), 25 column volumes of wash buffer-2 (20 mM Tris–HCl, pH 7.9, 500 mM NaCl, 20 mM imidazole), five column volumes of wash buffer-3 (20 mM Tris–HCl, pH 7.9, 500 mM NaCl, 50 mM imidazole) and five column volumes of wash buffer-4 (20 mM Tris–HCl, pH 7.9, 500 mM NaCl, 100 mM imidazole). Bound protein was eluted with 15 column volumes of elution buffer (20 mM Tris–HCl, pH 7.9, 500 mM NaCl, 300 mM imidazole).

Fractions containing significant amounts of purified protein, as examined by SDS–PAGE (12%) analysis were pooled and then concentrated to 250 µg/ml using Amicon Ultra-15 (5 K NMWL) (Millipore, USA) concentrator, and stored at 4°C. The concentration of protein was determined using bicinchoninic acid assay (BCA) (Bangalore Genei, India) method [45] using Bovine Serum Albumin (BSA) (Bangalore Genei, India) as standard. Identity of purified expressed recombinant was confirmed by peptide mass fingerprinting, in which spectral analysis of the tryptic digested peptides were searched using MASCOT search engine against MSDB (mass spectrometry protein sequence DataBase) [46].

### Western blotting

Samples were electrophoresed on 12% SDS-PAGE. Pre-stained molecular weight standards (10–170 kDa) (Fermentas, Canada) were co-electrophoresed for size determination. Electrophoresis was carried out at 100 V, 90 mA for 4–5 hours in Mini-PROTEAN Tetra Cell (Bio-Rad, USA). Proteins from the gel were transblotted onto nitrocellulose membranes using a Trans-Blot apparatus (Bio-Rad, USA) in transfer buffer (24 mM Tris-HCl (pH 8.0); 192 mM glycine; 20% methanol) for 2 hours at 90 V at 4°C. After transfer, blots were examined for transfer by Ponceau S (Sigma, USA) staining. Subsequently, blots were incubated in blocking buffer for 2 hours with PBS (137 mM NaCl, 2.7 mM KCl, 4.3 mM Na_2_HPO_4_, 1.47 mM KH_2_PO_4_) containing 5% Skimmed milk powder (Sigma, USA). After washing with PBS-Tween-20 (PBS-T) (0.2%) for 3 times and with PBS once, the blots were incubated with horseradish peroxidase (HRP) conjugated anti-histidine antibodies (Qiagen, Germany) at 1∶5000 dilution for three hours. The blots were further washed with PBS-T (0.2%) for 3 times and with PBS once and then developed with 3, 3′-Diaminobenzidine (DAB) (Sigma, USA).

### Circular Dichroism (CD)

JASCO J-815 CD Spectrometer was used to record CD spectra of purified recombinant N-terminal His-tagged protein at 0.1 mg/ml in 10 mM Potassium phosphate buffer, pH 7.4. Each spectrum was recorded at 1.0 nm intervals using a 0.5 cm path length cuvette. The spectra were taken in far-UV region from 190 to 260 nm and averaged over three scans. Recorded spectra in millidegrees of ellipticity were converted to ellipticity (θ) in deg. cm^2^ dmol^−1^ [47].

### Modified Enzyme Linked ImmunoSorbent Assay(ELISA)

Conventional ELISA on microtitre plate requires large quantities of analyte and reagents, which are precious and not available easily. Recently, an image- based ELISA has been developed on an activated polypropylene microtest plate (APPμTP), which is sensitive and requires 10 times less reagent than microtitre plate but has similar efficiency [48]. Polypropylene microtest plate has an array of small cavities made on a polypropylene sheet. In APPμTP, inert and hydrophobic cavities (test zones) were activated by a photolinker 1-fluoro-2-nitro-4-azidobenzene (FNAB) according to the published procedure [49]. The APPμTP so prepared can capture and immobilize a biomolecule through a covalent linkage thereby eliminating non-specific binding often prevalent in adsorption based techniques. We have used this modified ELISA on APPμTP to investigate the binding of the recombinant proteins with extracellular matrix proteins. Briefly, 200 ng of purified protein was covalently immobilized on an APPμTP by incubating for 12 hours at 25°C using 0.5 M carbonate bicarbonate buffer, pH 9.6. The following extracellular matrix proteins were immobilized: (i) Laminin from human fibroblasts (Sigma, USA), (ii) Fibronectin from human fibroblasts (Sigma,USA), (iii) Collagen from human placenta (Sigma, USA), (iv) purified recombinant protein as experimental positive control and (v) BSA (Sigma, USA) as negative protein control and (vi) Unrelated 6×Histidine tagged recombinant Mtb P-II (Rv2919c) protein (glnB), a nitrogen regulatory protein, as another Mtb protein negative control.

Plates were washed with PBS-T (0.2%) after each step. Blocking was carried out with 3% BSA for 2 hours. After washing the plates three times with PBS-T, each well was incubated with 200 ng of purified recombinant protein and incubated at 25°C for 3 hours. Plates were washed thrice with PBST and then incubated with 1∶1000 horseradish peroxidase (HRP) conjugated anti-6×Histidine antibodies (Qiagen, USA) for 3 hours. Finally, the color was developed adding ortho-phenylenediamine dihydrochloride (OPD) (Sigma, USA), followed by 5% H_2_SO_4_ to stop the color development. The plates were then scanned on a desktop scanner (HP photosmart C6388) to acquire the image. Image data was converted from red, green, and blue (RGB) scale to hue, saturation, and value (HSV) and then quantified as % saturation of color using in house R scripts. The significance of binding of adhesin protein with Laminin, Fibronectin, Collagen, BSA and Mtb PII were assessed by using Tukey-Kramer test in R using ‘DTK’ package [50]. Means of different groups were compared pairwise by controlling type 1 error to 1% and adjusted p-value<0.05.

### Gene Expression data analysis

We collected gene expression data from Gene Expression Omnibus (GEO) database for multiple conditions. These data on *M. tuberculosis* include whole genome expression of multiple strains under log phase of growth (GSE3201) [51], gene expression during adaptation to stationary phase and low oxygen dormancy (GSE8786) [52]; in vitro dormancy achieved by multiple stresses (GSE10391) [53]; transcriptional response to lung surfactants WLS (whole lung surfactants), PPL (column-purified surfactant lipids), CLSE (extracted lung surfactant) and SP-A (surfactant protein–A) (GSE14005) [54]; hypoxic and dosR mutation time course (GSE9331) [55]. We used data from 94 samples, including 10 multiple strains, 8 samples from exposure to lung surfactants (two time points; 30 minutes and 2 hours for each surfactant), 6 samples each of hypoxic response time course and of dosR mutant time course (4 hours, 8 hours, 12 hours, 1 day, 4 days and 7 days), 7 samples from multiple stresses (1 day, 2 days, 2 biological replicates of 3 days, 6 days, 9 days and 18 days), 3 samples from starved conditions (3 days, 9 days and 18 days) and 35 samples from oxygen depleted time course and 19 samples from stationary phase.

The log ratios (test vs. control) for all genes were taken together. All technical replicates were averaged. Expression values (log ratios) were normalized by using z-score transformation [56]. Z-score transforms each value after subtracting it from the sample mean and divided by standard deviation. After this transformation, mean of sample become zero and standard deviation (SD) become one (standard normal). Higher values of Z-score of log ratios correspond to high expression with respect to mean of expression of all genes in sample and higher expression with respect to the control. We categorized the expression of the genes in each case on the basis of Z-scores in 3 categories: high expression (Zscore> = 1), moderate expression (Zscore<1 and >−1) and low expression (Zscore<−1). We used gplots in R-package to create heatmaps [57].

## Results and Discussion

### Computational screening

A pipeline for work was designed (Figure 1A) to screen all proteins of *M. tuberculosis* through multiple available bioinformatic tools for identification of novel potential adhesins. Screening of all protein sequences from *M. tuberculosis* using SPAAN was carried out with a cutoff of P_ad_>0.65 because the well known adhesin HBHA had a P_ad_ value slightly lower than 0.7. Potential extracellular localization of these protein sequences were examined by LOCTree [27], PSORTb [28] and SubLoc [29]. After removing potential human homologues we obtained 68 proteins, of which 47 were globular proteins and 21 were non-globular proteins. Of these 68 proteins, subcellular localization tool LOCTree predicted 33 proteins to be extracellular. PSORTb predicted 15 proteins to be extracellular and 48 proteins could not be predicted for their sub-cellular localization and were given ‘unknown status’. SubLoc predicted 16 proteins to be extracellular. There were several cases wherein we couldn't reach a clear decision and therefore they were given ‘undecided’ status. The final result emerging from the decisions reached through argumentative approach (Figure 1B) and additional criteria such as preference for proteins of low molecular weight proteins and which have not been investigated previously are shown in Table 2.

**Table 2:** List of proteins meeting selection criteria, with high SPAAN score, globular proteins and have no reference in literature.

RvID	Protein*	Size (kDa)	SPAAN (P_ad_ Value)	Localization	Globular Proteins	CDD Domain Analysis (E Value)$
**Rv1116A**	Conserved hypothetical protein	9.040	0.717	Extracellular	Globular	None
**Rv1987**	Possible chitinase	14.920	0.670	Extracellular	Globular	Carbohydrate binding domain, Cellulose binding domain (4.69e-24)
**Rv1881c**	lppE (Lipoprotein)	14.940	0.664	Extracellular	Globular	Myco_19_kDa super family (6.82e-04)
**Rv2599**	Probable conserved membrane protein	14.950	0.752	Extracellular	Globular	Domain of unknown function (DUF4247) (2.07e-31)
**Rv1984c**	cfp21 (Hydrolyzes cutin)	21.780	0.685	Extracellular	Globular	Cutinase (2.20e-52)
**Rv0309**	Conserved exported protein	22.530	0.691	Extracellular	Globular	L,D-transpeptidase catalytic domain, YkuD, COG3786 (1.43e-79)
**Rv3576**	lppH (Possible conserved lipoprotein)	25.020	0.693	Extracellular	Globular	PknH-like extracellular domain (3.65e-47)
**Rv3822**	Conserved hypothetical protein	41.410	0.739	Extracellular	Globular	PE-PPE domain (1.53e-68)
**Rv0050**	ponA (Peptidoglycan synthesis)	71.120	0.688	Extracellular	Globular	Penicillin binding protein transpeptidase domain (1.41e-49)
**Rv0584**	Possible conserved exported protein	92.950	0.744	Extracellular	Globular	Glycosyl hydrolase family 92 (0e+00)
**Rv1037c**	esxI, (Putative ESAT-6 like protein)	9.830	0.737	Undecided	Globular	WXG100 (2.83e-08)
**Rv3890c**	esxC, (ESAT-6 like protein)	9.920	0.800	Undecided	Globular	COG4842[COG4842], Uncharacterized protein conserved in bacteria (1.65e-15)
**Rv3337**	Conserved hypothetical protein	13.180	0.698	Undecided	Globular	Abhydrolase_6[pfam12697], Alpha/beta hydrolase family (5.08e-04)
**Rv3067**	Conserved hypothetical protein	13.930	0.768	Undecided	Globular	Protein of unknown function (DUF732) pfam05305 (1.27e-19)
**Rv3705c**	Conserved protein	22.360	0.689	Undecided	Globular	PknH_C[pfam14032], PknH-like extracellular domain (6.95e-37)
**Rv1115**	Possible exported protein	24.050	0.665	Undecided	Globular	None
**Rv3717**	Conserved hypothetical protein	24.840	0.698	Undecided	Globular	MurNAc-LAA[cd02696], N-acetylmuramoyl-L-alanine amidase (8.86e-39)
**Rv0590**	mce2B, (MCE-Family protein)	29.160	0.685	Undecided	Globular	MCE[pfam02470], mce related protein, ABC-type transport system, (1.78e-47)
**Rv3207c**	Conserved protein	31.030	0.797	Undecided	Globular	DUF3152[pfam11350], Protein of unknown function (DUF3152) (2.07e-107)
**Rv0988** #	Possible conserved exported protein	42.780	0.697	Undecided	Globular	COG5621[COG5621], Predicted secreted hydrolase (2.01e-147)

Notes:

* Annotation according to TubercuList [42].

$ E-value from CDD search.

# There was no evidence from the algorithms for ‘extracellular’ (claim) for Rv0988. Therefore it was declared ‘undecided’ because the TubercuList annotation reports this protein as exported protein.

### Protein Expression and purification

As we began cloning and expression of the genes coding for the proteins listed in Table 2, we faced problems with expression and purification. Because these proteins are membrane associated, this might be somewhat expected. Despite various trials, which included use of *E. coli* strains capable of handling toxic proteins (C41 (DE3), C43 (DE3)), induction at various temperatures (16, 25, 30, 37°C), and purification under denaturation conditions, we were not successful in several cases. Based on good expression we could select Rv2599 and Rv0309. The protein Rv3717 with N-acetylmuramoyl-L-alanine amidase domain was selected from the list of ‘undecided’ group based on the literature search reports, which described its homologues in other pathogens as having role as adhesin [58]–[60]. We also succeeded in expressing Rv3717 in large amount. Based on these criteria and experiments we selected Rv2599, Rv0309 and Rv3717 for experimental analysis. The molecular features of these proteins are summarised in Table 3.

**Table 3:** Properties of selected proteins and their domain analysis.

Properties	Rv0309	Rv2599	Rv3717
**Molecular Size (kDa)**	22.52	14.95	24.80
**Number of Amino Acids**	218	143	241
**TMHMM**	1 (7–29)	1 (7–29)	0
**SignalP (Cleavage Site)**	Yes (34–35)	Yes (16–17)	Yes (24–25)
**Tuberculist Functional Annotation**	Possible conserved exported protein	Probable conserved membrane protein	Conserved hypothetical protein
**Functional Domains Present**	L,D-transpeptidase	DUF4247 domain	N-acetylmuramoyl-L-alanine amidase
**SPAAN Score**	0.691	0.752	0.698
**Globularity**	Globular	Globular	Globular

**Figure 2:**
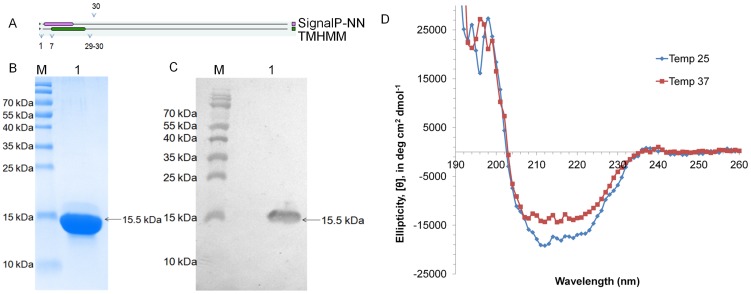
Purification and CD spectral analysis of rRv2599. A. Protein topology showing the presence of signal peptide and/or transmembrane helices in the protein signal peptide spans from 1–16 amino acids, whereas transmembrane domain spans from 7–29 amino acids. B. Purified protein run on 12% SDS-PAGE stained with coomassie. Lane M is molecular weight marker and Lane 1 is the purified recombinant rRv2599. C. Western blotting with Anti-His antibodies. Lane M is molecular weight marker and Lane 1 is the purified recombinant rRv2599. D. CD spectra of purified rRv2599 at two different temperatures 25°C and 37°C.

**Figure 3:**
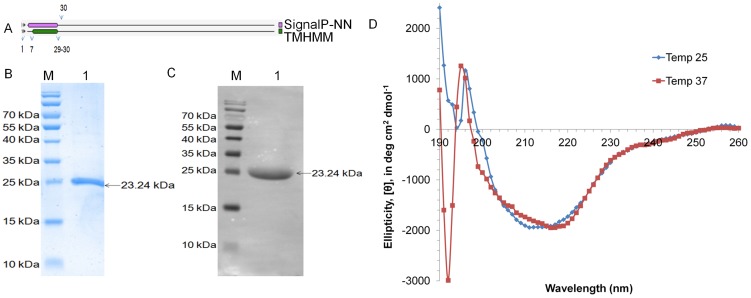
Purification and CD spectral analysis of rRv0309. A. Protein topology showing the presence of signal peptide and/or transmembrane helices in the protein signal peptide spans from 1–34 amino acids, whereas transmembrane domain spans from 7–29 amino acids. B. Purified protein run on 12% SDS-PAGE stained with coomassie. Lane M is molecular weight marker and Lane 1 is the purified recombinant rRv0309. C. Western blotting with Anti-His antibodies. Lane M is molecular weight marker and Lane 1 is the purified recombinant rRv0309. D. CD spectra of purified rRv0309 at two different temperatures 25°C and 37°C.

**Figure 4:**
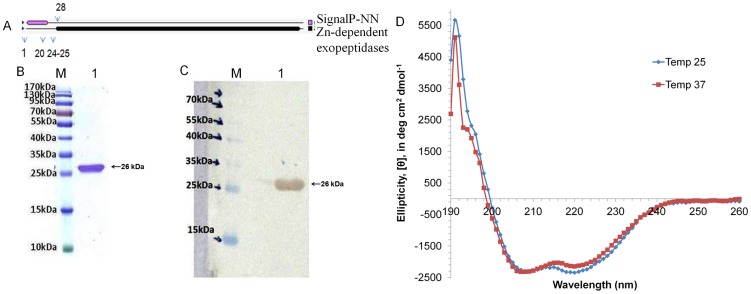
Purification and CD spectral analysis of rRv3717. A. Protein topology showing the presence of signal peptide and/or transmembrane helices in the protein. The signal peptide spans from 1–24 amino acids with cleavage site at 24–25 amino acids. B. Purified protein run on 12% SDS-PAGE stained with coomassie. Lane M is molecular weight marker and Lane 1 is the purified recombinant rRv3717. C. Western blotting with Anti-His antibodies. Lane M is molecular weight marker and Lane 1 is the purified recombinant rRv3717. D. CD spectra of purified rRv3717 at two different temperatures 25°C and 37°C.

The molecular features of the proteins show presence of signal peptide and transmembrane domain at N-terminus in Rv2599 (Figure 2A) and Rv0309 (Figure 3A). Rv3717 has signal peptide at the N-terminus (Figure 4A). Recombinant proteins after removal of signal peptide or transmembrane domain could be produced at good yields (∼2–3 mg/liter culture). The His-tagged recombinant proteins rRv2599, rRv0309 and rRv3717 had calculated molecular weight of 15.50 kDa, 23.24 kDa and 26.13 kDa respectively. In SDS-PAGE and western blot with anti-His antibodies we observed distinct bands at 15.5 kDa for rRv2599 (Figure 2B and 2C), at 23.5 kDa for rRv0309 (Figure 3B and 3C) and at 28 kDa for rRv3717 (Figure 4B and 4C) respectively, which closely correspond to the calculated molecular weights of the recombinant His-tagged proteins. As judged by Coomassie Blue-stained SDS-PAGE gels, we could achieve more than 99% pure recombinant proteins with no visible degradation. The Identities of the recombinant proteins were confirmed by peptide mass fingerprinting, using MALDI-TOF analysis. The peptides coverage obtained was 33.57%, 29.36% and 51.45% for Rv2599, Rv0309 and Rv3717 respectively.

### Rv2599

Domain analysis using Conserved Domain Database [34], Pfam [35] and InterProScan [36] showed presence of DUF4247 domain, a domain of unknown function. BLAST search revealed that Rv2599 is having homologues in Actinomycetales only and they are all annotated as hypothetical proteins. We could not identify sequence similarity of this protein to any protein with known function. No other protein with this domain has been reported in the literature as adhesin.

The far-UV CD spectra of purified rRv2599 taken at two different temperatures 25°C and 37°C are shown in Figure 2D. Dichroweb [61] analysis showed presence of structured protein with 39% alpha helix, 1% of beta sheets and 60% random coils.

### Rv0309

Searches with Domain database revealed presence of YkuD super family domain with L,D-transpeptidase catalytic domain in Rv0309. The YkuD domain consists of two beta-sheets forming a cradle capped by an alpha-helix [62]. The conserved region contains a conserved histidine and cysteine, with cysteine thought to be an active site residue [63].

The L,D-transpeptidases are widely distributed among bacterial species including Actinobacteria, Bacteroidetes, Firmicutes and Proteobacteria. The homologues of L,D-transpeptidases are well studied in *Bacillus subtilis* (ykuD) [62], *Enterococcus faecium* (Ldt_fm_) [64] and *E. coli* (ErfK, YcfS, YbiS, YcbB and YnhG) [63]. We searched for L,D-transpeptidases in UniProtKB database using the key word “L,D-transpeptidase” and obtained a total of 1,886 entries of which 21 were described as ‘reviewed’. All sequences were analyzed by SPAAN for identifying potential adhesin or adhesin-like proteins. Out of total 1,886 entries, 66 entries had P_ad_ value>0.65. None of the 21 reviewed entries had P_ad_ value>0.65. Our survey of literature did not show evidence of any L,D-transpeptidase that has been shown to be adhesin so far in any bacteria.

The CD spectrum of rRv0309 shows a typical β-sheet-rich structure (Figure 3D). Dichroweb [61] data analysis of the CD spectrum showed presence of 5% alpha helices, 47% beta sheet and 48% random coils in agreement with the general characteristics of proteins belonging to this family.

### Rv3717

Domain analysis showed that Rv3717 is a cell wall hydrolase with “N-acetylmuramoyl-L-alanine amidase” domain. Cell wall hydrolases were retrieved by searching the UniProtKB database with the term “N-acetylmuramoyl-L-alanine amidase”. There were a total of 20,900 entries, of which 135 were ‘reviewed’. These proteins are widely distributed among various organisms including those belonging to Archaea [65], Bacteria [66], Eukaryotes [67] and Viruses (Bacteriophages) [68]. All entries were scanned by SPAAN for their probability of being adhesin or adhesin-like proteins. Out of total 20,900 entries (135 were ‘reviewed). 4,765 were found to have P_ad_ value>0.65 and out of 135 reviewed, 44 entries, had P_ad_ value>0.65. Among these 44 entries highly similar protein sequences (with >95% identity) were removed resulting in 16 unique bacterial proteins. Among these, a few cell wall hydrolases were reported to have adhesin properties. These include AtlE protein, a cell wall hydrolase from *Staphylococcus epidermidis*, which mediates attachment to polystyrene, adherence to vitronectin, and participates in biofilm formation [69]. The Aaa from *Staphylococcus aureus* binds to fibrinogen, fibronectin and vitronectin dose-dependently and with high affinity [58]. The AtlE homologue, Aas from *Staphylococcus saprophyticus* [63] binds to fibronectin. In addition, the Aaa homologue Sle1 from *Staphylococcus saprophyticus* and *Staphylococcus epidermidis* also were shown to bind to fibronectin [59]. These reports show that some of the N-acetylmuramoyl-L-alanine amidases in pathogens have dual functions namely, as cell wall hydrolase and as adhesin.

**Figure 5:**
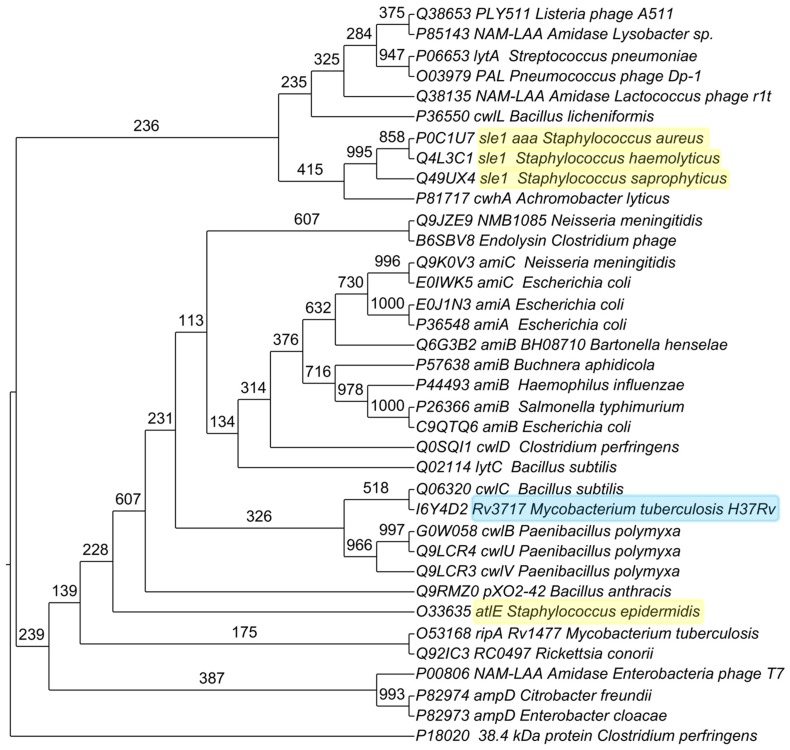
Phylogenetic analysis of Rv3717 and N-acetylmuramoyl-L-alanine amidase from various bacterial species. Proteins highlighted have N-acetylmuramoyl-L-alanine amidase activity and are adhesins. Numbers at fork are from bootstrap analysis.

The amino acid sequences of various N-acetylmuramoyl-L-alanine amidase homologues from several species were compared with Rv3717 and a phylogenetic tree was constructed from the distance matrix analysis (Figure 5). The Rv3717 groups with the cell wall hydrolase cwlC of *B. subtilis*. Other known cell wall hydrolases with adhesin properties form multiple groups even among the same genus *Staphylococcus*. It therefore appears that the adhesin properties of cell wall hydrolases have evolved in pathogens in multiple groups of species with different sequence relationships.

The CD spectrum of protein rRv3717 showed presence of 7% alpha helix, 47% beta sheets and 49% random coils (Figure 4D). Overall these proteins have structured conformation and therefore used for further investigations.

### Binding with extracellular matrix proteins

Pathogenic bacteria frequently express surface proteins with affinity for components of the mammalian extracellular matrix, namely collagens, laminin, fibronectin and proteoglycans [70]–[72]. It is therefore important to understand the role of components of bacterial cell surface that are involved in binding and interaction with the host extracellular matrix proteins.

**Figure 6:**
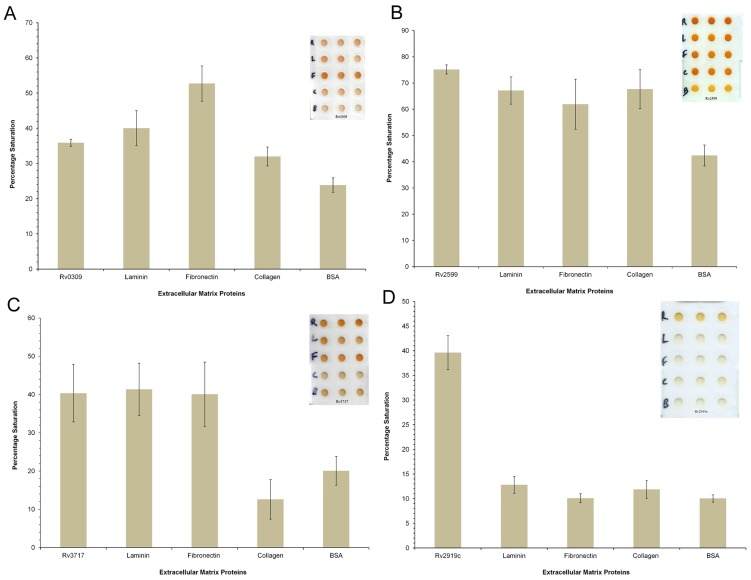
Binding activity of recombinant proteins with extracellular matrix proteins. Histograms of % color saturation with error bars showing 1 standard deviation (1 SD) of values obtained in triplicate experiments. A. rRv0309, B. rRv2599, C. rRv3717 and D. P-II nitrogen status signaling protein (rRv2919c).

The results of modified ELISA are shown in Figure 6. It is evident that the proteins rRv0309, rRv2599 and rRv3717 all showed propensity to bind to fibronectin (*P_adj_-value*<0.05), as good as positive control (Figure 6A, 6B and 6C). Under the same conditions His-tagged protein rRv2919c did not show binding to any of the extracellular matrix proteins (Figure 6D).

Fibronectin is a multidomain glycoprotein with multiple adhesive properties and it functions as a link between cells and their extracellular matrices. Fibronectin has major roles in adhesion, migration, differentiation and proliferation, and therefore, it is important in many physiological processes [73]. It is recognized to be target for large number of bacterial adhesins. This interaction appears to be crucial for establishment of infection in the host tissue, as is required for survival and replication [74]. It is reported that fibronectin facilitates attachment of *M. tuberculosis* to the host tissue [74]. However, the details of this attachment are not well known. In the last few years large numbers of bacterial fibronectin-binding proteins have been identified with role in virulence in both Gram-positive and Gram-negative bacteria [75].

Results of modified ELISA in the present study show that rRv0309, rRv2599 and rRv3717 bind to laminin in comparison to the negative controls (*P_adj_-value*<0.05) (Figure 6A, 6B and 6C). The ability of Rv0309 to bind to laminin is lower than that to fibronectin (*P_adj_-value*<0.05), but it is higher than that to collagen and to negative control (*P_adj_-value*<0.05). On the other hand both rRv2599 and rRv3717 bind to both fibronectin and laminin almost equally, in comparison to negative controls (*P_adj_-value*<0.05) (Figure 6B and 6C).

Extracellular matrix protein laminin is a large non-collagenous protein, which makes up the major part of basement membrane. The pathogenic bacteria use laminin as a mediator between bacteria and host cells [76]. Various proteins well established as adhesin binding to laminin are Lmb from *Streptococcus agalactiae* [77], Protein E from *Haemophilus influenza* [78], Protein F from *Haemophilus influenza* [79], AfCalAp from *Aspergillus fumigates* [24], 25-kDa outer membrane protein from *Helicobacter pylori* [80], Lbp from *Streptococcus pyogenes* [81], Hlp/LBP from *Mycobacterium leprae* [5], Lbp from *Streptococcus pyogenes* [81], Scl1 from *Streptococcus* [72], LBP from *M. tuberculosis* [7], Ace from *Enterococcus faecalis* [82], AlpA and AlpB from *Helicobacter pylori* [83], SabA from *Helicobacter pylori* [84], ErpX from *Borrelia burgdorferi* [85].

Both rRv0309 and rRv3717 did not show any binding to collagen. The recombinant protein rRv2599 shows significant binding with collagen in comparison to the negative controls (*P_adj_-value*<0.05) (Figure 6B). Collagens are widely found in all multicellular organisms with variety of functions, such as cell migration, cell adhesions, along with structural role in tissue integrity. It makes up a large component of extracellular matrix [86]. The ability of pathogens to adhere to collagen is advantageous for their pathogenesis [87], [88]. Several pathogenic bacterial species have been shown to be binding to collagen such as *Enterococcus faecalis* [89], *Psuedomonas aeruginosa* [90] and *Staphylococcus aureus* [91]. Some of the well known collagen binding adhesins are Acb from *Streptococcus gallolyticus* [92], YadA from *Yersinia enterocolitica* [93], CbpA from *Arcanobacterium pyogenes* [94], Cnm from *Streptococcus mutans* [95], 57-kDa collagen-binding protein from *Streptococcus pyogenes* [96], CAN from *Staphylococcus aureus* [97], GehD from *Staphylococcus epidermidis* [98], RspB from *Erysipelothrix rhusiopathiae* [99], Ace from *Enterococcus faecalis* [100], AhsA from *Mannheimia haemolytica* [101], cbsA from *Lactobacillus crispatus* [102].

Among the three new proteins we have characterized, Rv2599 had binding affinity with fibronectin, laminin and collagen. Based on structural features, the protein rRv2599 belongs to the class of adhesins with high (39%) alpha helix in comparison to beta sheets (1%). Adhesins with high alpha helix and low beta sheets though less in numbers, include Adhesion A (Q5I6B0) from *Fusobacterium nucleatum*, Fap1 adhesin (Fimbriae-associated protein Fap1) (A1C3L3) from *Streptococcus parasanguis*, Malate Synthase (P0A5J4) from *M. tuberculosis*, YadA (A1JUB7) from *Yersinia enterocolitica*, Hap from *Haemophilus influenza* [71], Lsa21 [103] and LigB from *Leptospira interrogans* [104]. LigB have been suggested to be useful for diagnosis and for vaccine candidate [104].

In general, adhesins whose crystal structures are available, are known to have low content of alpha helices average 5% (range 0–15%) and are mainly beta sheet rich proteins 47% (range 40–65%), a feature to which both Rv0309 and Rv3717 compare well. Adhesins with high beta sheet content include well established adhesins including pilus adhesin RRGA (2WW8) from *Streptococcus* pneu*m*oniae, YadA (2LME) from *Yersinia enterocolitica*, Hap adhesin (3SYJ) from *Haemophilus influenzae*, FimH (1KLF) from *E. coli* (strain K12), fimbrial adhesin FimA (3QDH) from *Streptococcus oralis*, FimF (3JWN) from *E. coli* (strain K12), Pilin (3GLE) from *Streptococcus pyogenes*, Invasin (4E1T) from *Yersinia pseudotuberculosis*, fnbpa-5 (3CAL) from *Staphylococcus aureus*.

### Gene Expression data analysis

**Figure 7:**
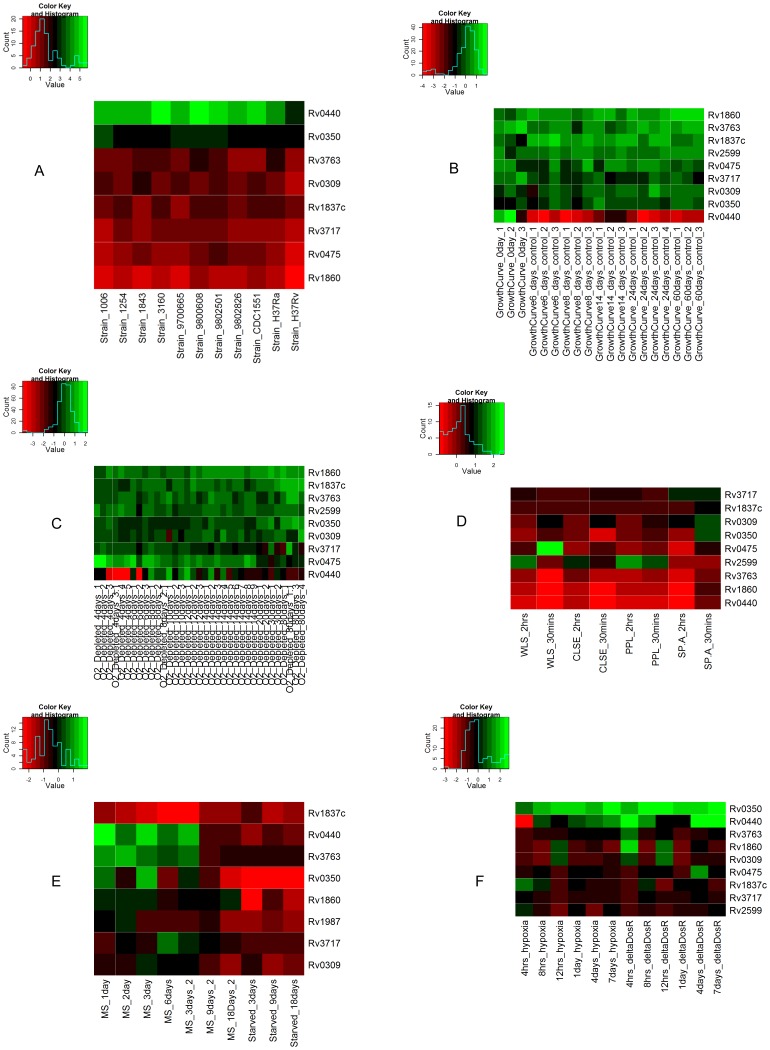
Heatmap of selected adhesin coding genes expressed in various experimental conditions in *M. tuberculosis*. Values are Z-scores of log (ratio) of averaged technical replicates of genes expressed in each condition. The color scale is shown against each heatmap.

To check the expression of predicted adhesin coding genes *Rv2599*, *Rv0309* and *Rv3717*, gene expression data from GEO were examined along with other known adhesins HBHA (*Rv0475*), 19-kDa antigen (*Rv3763*), malate synthase (*Rv1837c*), Apa (*Rv1860*), Cpn60.2 (*Rv0440*) and DnaK (Hsp70) (*Rv0350*) in exponential phase (mid log phase) across multiple strains, stationary phase, hypoxia and dosR mutant, exposure to lung surfactants, oxygen depletion and under multiple stresses. The Heatmap of this analysis is displayed in Figure 7.

In log phase growth, the genes Cpn60.2 *(Rv0440)*, Hsp70 *(Rv0350)*, malate synthase (*Rv1837c*), 19-kDa antigen (*Rv3763*), *Rv0309*, *Rv3717*, Apa *(Rv1860)* and HBHA (*Rv0475*) were expressed at levels between high and moderate (Figure 7A). The gene *Rv2599* is unexpressed in all strains in exponential phase [49].

In contrast to the log phase of growth, in stationary phase and oxygen depletion conditions, the Cpn60.*2* (*Rv0440*) had low expression (Figures 7B and 7C) and *Rv2599* is expressed moderately. Apa (*Rv1860*) had high expression in 60 days of stationary phase samples whereas moderate in other conditions. Hsp70 (*Rv0350*), HBHA (*Rv0475*), *Rv0309* and *Rv3717* were expressed moderately in most samples. The 19-kDa antigen (*Rv3763*) had moderate expression in most of the samples in stationary phase.

In the case of exposure to lung surfactants (Figure 7D), *Rv3717* showed high expression with SP-A and moderate expression in all other samples. *Rv2599* had high expression in 2 hours of WLS exposure and 2 hours of CLSE exposure and in 30 minutes and 2 hours exposure to PPL. *Rv0309* and Hsp70 (*Rv0350*) had high expression with 30 minutes exposure to SP-A. All other genes in all samples had moderate expression.

In the case of multiple stress conditions (Figure 7E), *Rv2599*, *Rv3717*, 19-kDa antigen (*Rv3763*) and Apa (*Rv1860*) had low to moderate expression in multiple stress conditions whereas malate synthase (*Rv1837c*) had low expression in most of the samples. *Rv0309* had moderate expression in 1,2,3,6 and 9 days of multiple stress treatment and low in starved conditions. Other genes Cpn60.*2 (Rv0440)* and Hsp70 *(Rv0350)* had low to high expression across different stress conditions.

In the cases of hypoxia and dosR mutants (Figure 7F), Hsp70 (*Rv0350*) had high expression in all samples of hypoxia and dosR mutants. *Rv2599*, *Rv3717*, 19-kDa antigen (*Rv3763*), malate synthase (*Rv1837c*), HBHA (*Rv0475*) and *Rv0309* had moderate expression in both hypoxia and dosR mutants across most of the time points. Cpn60.2 (*Rv0440*) and Apa (*Rv1860*) had low to high expression in hypoxia and dorR mutant time course.

It is evident that some adhesins (e.g. Cpn60.2) are expressed at high levels compared to others in the log phase of growth whereas in other conditions of stationary phase or other forms of stress, these genes are expressed at low levels. Concomitantly, the expression of other adhesins increase. The expression of some adhesins like *Rv2599*, increase significantly compared with other genes with exposure to lung surfactants indicating that these adhesins might be participating in interaction with the host cells. It was shown previously that bacterial cells become more adhesive in stationary phase and therefore this phase of growth stage might be accompanied by expression of other adhesin genes [105].

The adhesin function in host pathogen interaction is critical for survival of pathogen within the host. Multiple adhesins have been implicated in numerous pathogens [106] and the expression of the corresponding genes is also tightly regulated [107]. In several instances adhesins were usually identified using sequence similarity approach or using a defined model for bacterial cell-host cell interaction. In this work we used an integrative approach of the initial steps based on reverse vaccinology, including computational algorithms SPAAN and subcellular localization, which are widely used for many pathogens to identify putative adhesins [24]–[26], [108]. Using this approach we could identify a few novel adhesins in *M. tuberculosis*, which show potential to bind to some components of the host extracellular matrix.

**Figure 8:**
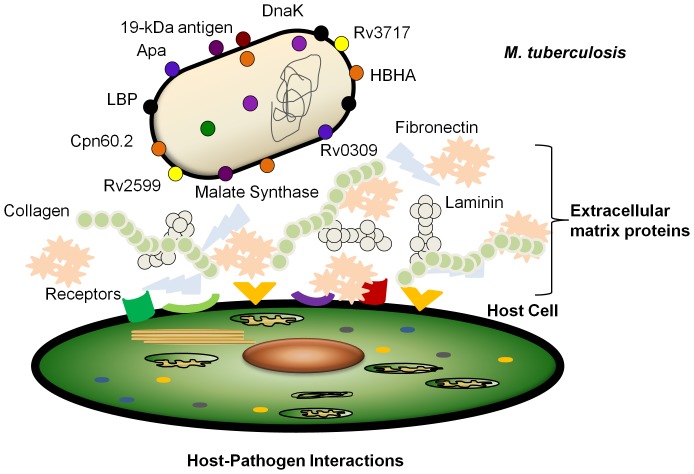
System of various adhesive molecules having role in adherence of *M. tuberculosis* to the host. The pathogen alters its surface adhesins through regulation of gene expression under different conditions of stress faced during its pathogenic life cycle.

The role of cell wall hydrolases also having functional role of adhesins is similar to the cases of AtlE from *Staphylococcus epidermidis* [69], Aaa from *Staphylococcus aureus* [58], Aas from *Staphylococcus saprophyticus* [60], Aaa (Sle1) from *Staphylococcus saprophyticus* [59]. The enzymatic activity of Rv3717 is also characterized (unpublished data) and its crystal structure was also determined (unpublished data, PDB-4HJN). The data confirm the enzymatic activity of Rv3717 as cell wall hydrolase, specifically, N-acetylmuramoyl-L-alanine amidase. Therefore, Rv3717 has both enzymatic activity of cell wall hydrolysis and adhesin function. Another enzyme malate synthase was also reported recently to have dual functions, namely the enzymatic activity of malate synthase and adhesin function [10]. The Rv0309 may also fit in this category although its enzymatic activity remains to be established. Proteins with more than one physiological function are reported by various authors and are termed as ‘moonlighting proteins’ [109], which are attracting attention recently. The microarray data shows that the expression of the system of adhesin coding genes of *M. tuberculosis* is also regulated differently under different conditions of interaction with the host (Figure 8). We propose a systemic approach to investigate the roles of various adhesins in the infection of *M. tuberculosis* that may enable better selection of potential vaccine candidates.

## Supporting Information

Table S1 List of *M. tuberculosis* proteins with SPAAN score>0.65 and human homologues removed. (DOC)
