# Supplementary material for: Identification of Novel Adhesins of M. tuberculosis H37Rv Using Integrated Approach of Multiple Computational Algorithms and Experimental Analysis
Source: PLoS One. 2013 Jul 29;8(7):e69790. doi: 10.1371/journal.pone.0069790 (PMC3726780; doi:10.1371/journal.pone.0069790)
Supplement: Table S1 — (DOC) [file pone.0069790.s001.doc]

**Table S1. List of *M. tuberculosis* proteins with SPAAN score > 0.65 and human homologues removed.**

| RvID | Protein^$^ | Size (kDa) | SPAAN | LOCTree* | PSORTb* | SubLoc* | Localization | Globular/Non | References |
| --- | --- | --- | --- | --- | --- | --- | --- | --- | --- |
| Rv0050 | ponA (Peptidoglycan synthesis) | 71.12 | 0.688 | E 2 | E 9.55 | P 4 | Claim (Extracellular) | Globular | No Reference |
| Rv0109 | PE-PGRS family protein (PE_PGRS1) | 42.29 | 0.832 | P 9 | E 9.73 | E 10 | Claim (Extracellular) | Non Globular | No Reference |
| Rv0124 | PE-PGRS family protein (PE_PGRS2) | 40.89 | 0.806 | P 5 | E 9.55 | E 10 | Claim (Extracellular) | Non Globular | No Reference |
| Rv0159c | PE family protein PE3 | 48.61 | 0.781 | C 9 | U 0 | P 3 | No Claim | Non Globular | No Reference |
| Rv0169 | MCE-family protein MCE1A | 47.79 | 0.692 | E 6 | P 9.26 | P 2 | No Claim | Non Globular | Yes [1,2] |
| Rv0287 | ESAT-6 like protein ESXG (Conserved protein TB9.8) | 9.78 | 0.713 | E 6 | U 0 | P 8 | No Claim | Non Globular | Yes [3,4] |
| Rv0288 | esxH, cfp7 TB10.4 | 10.39 | 0.660 | E 10 | U 0 | C 3 | Claim (Extracellular) | Globular | Yes [4,5] |
| Rv0309 | Conserved exported protein | 22.53 | 0.691 | E 6 | E 10 | P 3 | Claim (Extracellular) | Globular | No Reference |
| Rv0354c | PPE family protein PPE7 | 14.39 | 0.782 | P 6 | U 0 | C 1 | No Claim | Non Globular | No Reference |
| Rv0455c | Conserved protein | 16.64 | 0.678 | E 2 | U 0 | E 3 | Undecided | Globular | Yes [6] |
| Rv0584 | Possible conserved exported protein | 92.95 | 0.744 | E 6 | E 9.04 | E 3 | Claim (Extracellular) | Globular | No Reference |
| Rv0590 | mce2B, (MCE-family protein MCE2B) | 29.16 | 0.685 | P 5 | U 0 | C 5 | Undecided | Globular | No Reference |
| Rv0988^#^ | Possible conserved exported protein | 42.78 | 0.697 | P 10 | U 0 | P 1 | Undecided | Globular | No Reference |
| Rv1037c | esxI, Putative ESAT-6 like protein ESXI | 9.83 | 0.737 | C 2 | U 0 | C 3 | Undecided | Globular | No Reference |
| Rv1087 | PE-PGRS family protein (PE_PGRS21) | 62.47 | 0.875 | P 4 | E 9.97 | P 1 | No Claim | Non Globular | No Reference |
| Rv1089 | PE family protein (PE10) | 11.76 | 0.741 | E 10 | E 8.82 | E 7 | Claim (Extracellular) | Non Globular | No Reference |
| Rv1115 | Possible exported protein | 24.05 | 0.665 | E 6 | U 0 | P 4 | Undecided | Globular | No Reference |
| Rv1116A | Conserved hypothetical protein | 9.04 | 0.717 | E 10 | U 0 | E 10 | Claim (Extracellular) | Globular | No Reference |
| Rv1195 | PE family protein PE13 | 9.58 | 0.780 | E 6 | E 8.82 | P 10 | Claim (Extracellular) | Non Globular | Yes [7] |
| Rv1196 | PPE family protein PPE18 | 39.16 | 0.776 | P 6 | U 0 | P 3 | No Claim | Non Globular | Yes [8,9] |
| Rv1198 | esxL (ES6_4 Mtb9.9C , ESAT-6 like protein ) | 9.93 | 0.751 | C 2 | U 0 | P 2 | No Claim | Globular | Yes [10,11] |
| Rv1269c | Conserved probable secreted protein | 12.55 | 0.674 | P 10 | U 0 | P 3 | No Claim | Non Globular | No Reference |
| Rv1271c | Conserved hypothetical secreted protein | 11.59 | 0.797 | P 5 | U 0 | P 4 | No Claim | Globular | Yes [12] |
| Rv1291c | Conserved hypothetical secreted protein | 11.03 | 0.724 | P 5 | U 0 | P 5 | No Claim | Non Globular | No Reference |
| Rv1362c | Possible membrane protein | 23.47 | 0.676 | C 2 | U 0 | P 4 | No Claim | Globular | No Reference |
| Rv1690 | Probable lipoprotein LPRJ | 13.11 | 0.711 | E5 | P 9.26 | C 1 | No Claim | Globular | Yes [13] |
| Rv1791 | PE family protein PE19 | 9.58 | 0.674 | E 6 | E 8.82 | P 8 | Claim (Extracellular) | Non Globular | No Reference |
| Rv1881c | lppE (Lipoprotein) | 14.94 | 0.664 | E 2 | U 0 | E 1 | Claim (Extracellular) | Globular | No Reference |
| Rv1891 | Conserved protein | 14.11 | 0.713 | P 5 | U 0 | P 2 | No Claim | Globular | No Reference |
| Rv1906c | Conserved protein | 15.54 | 0.761 | P 5 | U 0 | P 8 | No Claim | Globular | No Reference |
| Rv1980c | mpt64, Immunogenic protein MPT64 (ANTIGEN MPT64/MPB64) | 24.82 | 0.687 | P 9 | U 0 | P 5 | No Claim | Globular | Yes [14,15] |
| Rv1984c | cfp21 (Hydrolyzes cutin) | 21.78 | 0.685 | E 6 | U 0 | E 3 | Claim (Extracellular) | Globular | No Reference |
| Rv1987 | Possible Chitinase | 14.92 | 0.670 | E 10 | E 9.73 | E 5 | Claim (Extracellular) | Globular | No Reference |
| Rv2046 | lppI, Probable lipoprotein | 22.20 | 0.732 | E 2 | U 0 | C 1 | Undecided | Non Globular | No Reference |
| Rv2077A | Conserved hypothetical protein | 9.61 | 0.660 | E 6 | U 0 | P 3 | Claim (Extracellular) | Non Globular | No Reference |
| Rv2341 | Probable conserved lipoprotein LPPQ | 14.14 | 0.826 | P 5 | U 0 | P 3 | No Claim | Globular | No Reference |
| Rv2346c | esxO, putative ESAT-6 like protein ESXO | 9.95 | 0.688 | C 2 | C 8.87 | C 6 | No Claim | Globular | Yes [16,17] |
| Rv2376c | cfp2, Low molecular weight antigen | 16.64 | 0.763 | P 6 | E 10 | P 3 | No Claim | Globular | Yes [18,19] |
| Rv2389c | rpfD, Probable resuscitation-promoting factor | 15.68 | 0.715 | E 6 | U 0 | P 3 | Claim (Extracellular) | Globular | Yes [20,21] |
| Rv2599 | Probable conserved membrane protein | 14.95 | 0.752 | E 6 | U 0 | E 8 | Claim (Extracellular) | Globular | No Reference |
| Rv2641 | cadI, Cadmium inducible protein | 16.03 | 0.695 | C 2 | U 0 | P 2 | Undecided | Globular | Yes [22] |
| Rv3019c | esxR, secreted ESAT-6 like protein ESXR (TB10.3) | 10.31 | 0.663 | E 2 | U 0 | E 1 | Claim (Extracellular) | Globular | Yes [3,23] |
| Rv3020c | esxS, ESAT-6 like protein | 9.84 | 0.714 | E 6 | U 0 | P 7 | Undecided | Non Globular | Yes [4,16,23] |
| Rv3067 | Conserved hypothetical protein | 13.93 | 0.768 | E 6 | U 0 | P 4 | Undecided | Globular | No Reference |
| Rv3097c | lipY, PE_PGRS63 | 44.99 | 0.679 | C 2 | U 0 | P 4 | Undecided | Globular | Yes [24,25] |
| Rv3207c | Conserved protein | 31.03 | 0.797 | C 5 | U 0 | P 4 | Undecided | Globular | No Reference |
| Rv3221c | Biotinylated protein TB7.3 | 7.31 | 0.227 | P 9 | C 8.87 | C 10 | Undecided | Globular | Yes [26] |
| Rv3337 | Conserved hypothetical protein | 13.18 | 0.698 | P 6 | U 0 | E 5 | Undecided | Globular | No Reference |
| Rv3369 | Conserved protein | 15.72 | 0.687 | C 2 | C 8.87 | P 1 | No Claim | Globular | Yes [27] |
| Rv3477 | PE family protein PE31 | 9.76 | 0.687 | E 6 | U 0 | P 4 | Undecided | Non Globular | No Reference |
| Rv3512 | PE_PGRS56, PE-PGRS family protein | 81.16 | 0.895 | P 10 | E 9.97 | E 10 | Claim (Extracellular) | Non Globular | No Reference |
| Rv3572 | Unknown protein | 18.74 | 0.716 | P 5 | U 0 | P 7 | No Claim | Globular | No Reference |
| Rv3576 | lppH, possible conserved lipoprotein | 25.02 | 0.693 | E 6 | U 0 | P 2 | Claim (Extracellular) | Globular | No Reference |
| Rv3584 | lpqE, possible conserved lipoprotein | 18.79 | 0.685 | P 5 | U 0 | P 2 | No Claim | Globular | No Reference |
| Rv3613c | Hypothetical protein | 5.61 | 0.670 | E 6 | U 0 | P 3 | Claim (Extracellular) | Globular | Yes [28,29] |
| Rv3619c | esxV, putative ESAT-6 like protein | 9.83 | 0.737 | C 2 | U 0 | C 3 | No Claim | Globular | No Reference |
| Rv3622c | PE family protein PE32 | 9.70 | 0.711 | E 6 | U 0 | C 3 | Claim (Extracellular) | Non Globular | No Reference |
| Rv3705c | Conserved protein | 22.36 | 0.689 | C 3 | U 0 | P 1 | Undecided | Globular | No Reference |
| Rv3717 | Conserved hypothetical protein | 24.84 | 0.698 | C 2 | U 0 | E 3 | Undecided | Globular | No Reference |
| Rv3724B | cut5b, Probable cutinase | 18.76 | 0.690 | E 8 | U 0 | P 1 | Claim (Extracellular) | Non Globular | No Reference |
| Rv3803c | fbpD, secreted MPT51/MPB51 antigen protein (fibronectin-binding protein C) (85C) | 31.09 | 0.692 | E 6 | E 10 | E 7 | Claim (Extracellular) | Globular | Yes [22,30] |
| Rv3804c | fbpA, secreted antigen 85-A (Fibronectin-binding protein A) (ANTIGEN 85 COMPLEX A) | 35.69 | 0.711 | E 6 | E 10 | E 1 | Claim (Extracellular) | Globular | Yes [31,32] |
| Rv3822 | Conserved hypothetical protein | 41.41 | 0.739 | E 6 | U 0 | P 3 | Claim (Extracellular) | Globular | No Reference |
| Rv3865 | espF, ESX-1 Secretion-associated protein | 10.62 | 0.772 | E 6 | U 0 | P 3 | Claim (Extracellular) | Globular | Yes [27,33] |
| Rv3875 | esxA, 6 KDA Early secretory antigenic target ESXA (ESAT-6) | 9.90 | 0.809 | E 6 | E 10 | C 1 | Claim (Extracellular) | Globular | Yes [34,35] |
| Rv3880c | espL, ESX-1 Secretion-associated protein | 12.17 | 0.671 | C 8 | U 0 | C 4 | No Claim | Non Globular | No Reference |
| Rv3890c | esxC, ESAT-6 like protein | 9.92 | 0.800 | P 1 | U 0 | C 1 | Undecided | Globular | No Reference |
| Rv3891c | esxD, Possible ESAT-6 like protein | 11.16 | 0.697 | P 5 | U 0 | C 3 | No Claim | Globular | No Reference |

Notes:

^$^ Annotation according to TubercuList.

*E – Extracellular localization, C – Cytoplasmic localization and U – Unknown localization. Numbers given along the predictions is the confidence of prediction in three categories: 0-3, low; 3-6, medium; > 6, high.

^#^ There was no evidence from the algorithms for ‘extracellular’ (claim) for this protein. Therefore it was declared ‘undecided’ because the TubercuList annotation reports this protein as exported protein.

Supplementary References

1. Saini NK, Sharma M, Chandolia A, Pasricha R, Brahmachari V, et al. (2008) Characterization of Mce4A protein of *Mycobacterium tuberculosis*: role in invasion and survival. BMC Microbiol 8: 200.

2. Pasricha R, Chandolia A, Ponnan P, Saini NK, Sharma S, et al. (2011) Single nucleotide polymorphism in the genes of mce1 and mce4 operons of *Mycobacterium tuberculosis*: analysis of clinical isolates and standard reference strains. BMC Microbiol 11: 41.

3. Lightbody KL, Renshaw PS, Collins ML, Wright RL, Hunt DM, et al. (2004) Characterisation of complex formation between members of the *Mycobacterium tuberculosis* complex CFP-10/ESAT-6 protein family: towards an understanding of the rules governing complex formation and thereby functional flexibility. FEMS Microbiol Lett 238: 255-262.

4. Lightbody KL, Ilghari D, Waters LC, Carey G, Bailey MA, et al. (2008) Molecular features governing the stability and specificity of functional complex formation by *Mycobacterium tuberculosis* CFP-10/ESAT-6 family proteins. J Biol Chem 283: 17681-17690.

5. Ilghari D, Lightbody KL, Veverka V, Waters LC, Muskett FW, et al. (2011) Solution structure of the *Mycobacterium tuberculosis* EsxG.EsxH complex: functional implications and comparisons with other M. tuberculosis Esx family complexes. J Biol Chem 286: 29993-30002.

6. Olsen I, Reitan LJ, Wiker HG (2000) Distinct differences in repertoires of low-molecular-mass secreted antigens of Mycobacterium avium complex and *Mycobacterium tuberculosis*. J Clin Microbiol 38: 4453-4458.

7. Goldstone RM, Goonesekera SD, Bloom BR, Sampson SL (2009) The transcriptional regulator Rv0485 modulates the expression of a pe and ppe gene pair and is required for *Mycobacterium tuberculosis* virulence. Infect Immun 77: 4654-4667. IAI.01495-08 [pii];10.1128/IAI.01495-08 [doi].

8. Nair S, Ramaswamy PA, Ghosh S, Joshi DC, Pathak N, et al. (2009) The PPE18 of *Mycobacterium tuberculosis* interacts with TLR2 and activates IL-10 induction in macrophage. J Immunol 183: 6269-6281.

9. Nair S, Pandey AD, Mukhopadhyay S (2011) The PPE18 protein of *Mycobacterium tuberculosis* inhibits NF-kappaB/rel-mediated proinflammatory cytokine production by upregulating and phosphorylating suppressor of cytokine signaling 3 protein. J Immunol 186: 5413-5424.

10. Bukka A, Price CT, Kernodle DS, Graham JE (2011) *Mycobacterium tuberculosis* RNA Expression Patterns in Sputum Bacteria Indicate Secreted Esx Factors Contributing to Growth are Highly Expressed in Active Disease. Front Microbiol 2: 266.

11. Mattow J, Schaible UE, Schmidt F, Hagens K, Siejak F, et al. (2003) Comparative proteome analysis of culture supernatant proteins from virulent *Mycobacterium tuberculosis* H37Rv and attenuated M. bovis BCG Copenhagen. Electrophoresis 24: 3405-3420.

12. Ben AY, Shashkina E, Johnson S, Bifani PJ, Kurepina N, et al. (2005) Immunological characterization of novel secreted antigens of *Mycobacterium tuberculosis*. Scand J Immunol 61: 139-146.

13. Steyn AJ, Joseph J, Bloom BR (2003) Interaction of the sensor module of *Mycobacterium tuberculosis* H37Rv KdpD with members of the Lpr family. Mol Microbiol 47: 1075-1089.

14. Mustafa AS (2010) In silico binding predictions for identification of HLA-DR-promiscuous regions and epitopes of *Mycobacterium tuberculosis* protein MPT64 (Rv1980c) and their recognition by human Th1 cells. Med Princ Pract 19: 367-372.

15. Danahy JM, Potter BM, Geisbrecht BV, Laity JH (2005) NMR assignment of protein Rv1980c from *Mycobacterium tuberculosis*. J Biomol NMR 33: 73.

16. Jones GJ, Hewinson RG, Vordermeier HM (2010) Screening of predicted secreted antigens from Mycobacterium bovis identifies potential novel differential diagnostic reagents. Clin Vaccine Immunol 17: 1344-1348.

17. He XY, Zhuang YH, Zhang XG, Li GL (2003) Comparative proteome analysis of culture supernatant proteins of *Mycobacterium tuberculosis* H37Rv and H37Ra. Microbes Infect 5: 851-856. S1286457903001795 [pii].

18. Zhang G, Zhang L, Zhang M, Pan L, Wang F, et al. (2009) Screening and assessing 11 *Mycobacterium tuberculosis* proteins as potential serodiagnostical markers for discriminating TB patients from BCG vaccinees. Genomics Proteomics Bioinformatics 7: 107-115.

19. Harboe M, Wiker HG (1998) Secreted proteins of Mycobacterium leprae. Scand J Immunol 48: 577-584.

20. Romano M, Aryan E, Korf H, Bruffaerts N, Franken CL, et al. (2012) Potential of *Mycobacterium tuberculosis* resuscitation-promoting factors as antigens in novel tuberculosis sub-unit vaccines. Microbes Infect 14: 86-95.

21. Commandeur S, van Meijgaarden KE, Lin MY, Franken KL, Friggen AH, et al. (2011) Identification of human T-cell responses to *Mycobacterium tuberculosis* resuscitation-promoting factors in long-term latently infected individuals. Clin Vaccine Immunol 18: 676-683.

22. Hotter GS, Wilson T, Collins DM (2001) Identification of a cadmium-induced gene in Mycobacterium bovis and *Mycobacterium tuberculosis*. FEMS Microbiol Lett 200: 151-155. S0378109701002142 [pii].

23. Arbing MA, Kaufmann M, Phan T, Chan S, Cascio D, et al. (2010) The crystal structure of the *Mycobacterium tuberculosis* Rv3019c-Rv3020c ESX complex reveals a domain-swapped heterotetramer. Protein Sci 19: 1692-1703.

24. Singh VK, Srivastava V, Singh V, Rastogi N, Roy R, et al. (2011) Overexpression of Rv3097c in *Mycobacterium bovis* BCG abolished the efficacy of BCG vaccine to protect against *Mycobacterium tuberculosis* infection in mice. Vaccine 29: 4754-4760.

25. Cascioferro A, Delogu G, Colone M, Sali M, Stringaro A, et al. (2007) PE is a functional domain responsible for protein translocation and localization on mycobacterial cell wall. Mol Microbiol 66: 1536-1547.

26. Kumar N, Shukla S, Kumar S, Suryawanshi A, Chaudhry U, et al. (2008) Intrinsically disordered protein from a pathogenic mesophile *Mycobacterium tuberculosis* adopts structured conformation at high temperature. Proteins 71: 1123-1133. 10.1002/prot.21798 [doi].

27. Bahk YY, Kim SA, Kim JS, Euh HJ, Bai GH, et al. (2004) Antigens secreted from *Mycobacterium tuberculosis*: identification by proteomics approach and test for diagnostic marker. Proteomics 4: 3299-3307.

28. Hunt DM, Sweeney NP, Mori L, Whalan RH, Comas I, et al. (2012) Long-range transcriptional control of an operon necessary for virulence-critical ESX-1 secretion in *Mycobacterium tuberculosis*. J Bacteriol 194: 2307-2320.

29. Rickman L, Scott C, Hunt DM, Hutchinson T, Menendez MC, et al. (2005) A member of the cAMP receptor protein family of transcription regulators in *Mycobacterium tuberculosis* is required for virulence in mice and controls transcription of the rpfA gene coding for a resuscitation promoting factor. Mol Microbiol 56: 1274-1286.

30. Ramalingam B, Baulard AR, Locht C, Narayanan PR, Raja A (2004) Cloning, expression, and purification of the 27 kDa (MPT51, Rv3803c) protein of *Mycobacterium tuberculosis*. Protein Expr Purif 36: 53-60.

31. Parker SK, Barkley RM, Rino JG, Vasil ML (2009) *Mycobacterium tuberculosis* Rv3802c encodes a phospholipase/thioesterase and is inhibited by the antimycobacterial agent tetrahydrolipstatin. PLoS One 4: e4281.

32. Alvarez-Corrales N, Ahmed RK, Rodriguez CA, Balaji KN, Rivera R, et al. (2013) Differential cellular recognition pattern to M. tuberculosis targets defined by IFN-gamma and IL-17 production in blood from TB + patients from Honduras as compared to health care workers: TB and immune responses in patients from Honduras. BMC Infect Dis 13: 125.

33. Brodin P, Majlessi L, Marsollier L, de Jonge MI, Bottai D, et al. (2006) Dissection of ESAT-6 system 1 of *Mycobacterium tuberculosis* and impact on immunogenicity and virulence. Infect Immun 74: 88-98.

34. Zeng J, Zhang L, Li Y, Wang Y, Wang M, et al. (2010) Over-producing soluble protein complex and validating protein-protein interaction through a new bacterial co-expression system. Protein Expr Purif 69: 47-53.

35. Hanif SN, Al-Attiyah R, Mustafa AS (2010) Molecular cloning, expression, purification and immunological characterization of three low-molecular weight proteins encoded by genes in genomic regions of difference of *Mycobacterium tuberculosis*. Scand J Immunol 71: 353-361.
